# Predictive value of homocysteine for depression after acute coronary syndrome

**DOI:** 10.18632/oncotarget.11966

**Published:** 2016-09-10

**Authors:** Hee Ju Kang, Robert Stewart, Kyung Yeol Bae, Sung Wan Kim, Il Seon Shin, Hyuno Kang, Won Jin Moon, Young Joon Hong, Youngkeun Ahn, Myung Ho Jeong, Jin Sang Yoon, Jae Min Kim

**Affiliations:** ^1^ Department of Psychiatry, Chonnam National University Medical School, Gwangju, Korea; ^2^ King's College London, Institute of Psychiatry, London, UK; ^3^ Gwangju Center, Korea Basic Science Institute, Gwangju, Korea; ^4^ Department of Cardiology, Chonnam National University Medical School, Gwangju, Korea

**Keywords:** depression, homocysteine, MTHFR C677T polymorphism, acute coronary syndrome, biomarkers

## Abstract

We investigated roles of plasma homocysteine and MTHFR gene in relation to risks and treatment responses of depression in ACS. A sample of 969 patients with recent ACS were recruited and 711 followed 1 year later. In addition, of 378 baseline participants with depressive disorder, 255 were randomized to a 24-week double blind trial of escitalopram (*N* = 127) or placebo (*N* = 128). A higher homocysteine concentration was independently associated with prevalent depressive disorder at baseline irrespective of MTHFR genotype; and with both incident and persistent depressive disorder at follow-up only in the presence of TT genotype. MTHFR genotype was not itself associated with depressive disorder after ACS. No associations were found with 24-week antidepressant treatment responses. Plasma homocysteine could be a biomarker for depressive disorder particularly in the acute phase of ACS. Focused interventions for those with higher homocysteine level and MTHFR TT genotype might reduce the risk of later depressive disorder.

## INTRODUCTION

Depression is commonly comorbid with acute coronary syndrome (ACS) [1, 2], with multiple biomarkers suggested as potential explanations [3-5]. Homocysteine is a potential candidate, since it has been associated with both disorders [4]. Homocysteine can cause atherothrombosis by various insults on vascular system, and therefore increase the risk for ACS [6], as well as directly inhibiting monoamine neurotransmitter metabolism and thus linked with depression [7, 8]. Whether homocysteine is a common biomarker for ACS and depression has not yet been investigated.

Homocysteine level are influenced complex systems including nutritional deficiencies or supplement of vitamins, and medical conditions such as chronic kidney disease, diabetes, and malignancies [9]. Homocysteine levels are also strongly influenced by genetic factors particularly for methylenetetrahydrofolate reductase (MTHFR), which catalyzes the reduction of 5,10-methylenetetrahydrofolate to 5-methyltetrahydrofolate. A 677C->T variant of MTHFR gene is responsible for thermolabile MTHFR with reduced enzymatic activity [10], and therefore and T allele is associated with higher homocysteine level [11, 12]. Relevant to these findings, the T allele of MTHFR gene has been reported to be associated with the ACS [13] and depression susceptibility [14]. However, it has not been investigated as a risk factor for depression occurring in ACS.

With respect to treatment response, homocysteine and MTHFR C677T polymorphism have additionally been investigated as predictors of antidepressant response in patients with major depressive disorder [15, 16], but not to date in ACS.

Using the data from observational prospective study in Koreans patients with ACS, this study aimed to investigate the roles of plasma homocysteine levels and MTHFR C677T polymorphism in relation to risks and treatment responses of depression in ACS.

## RESULTS

### Recruitment

The recruitment process is described in Supplemental Figure S1. Of the total baseline sample (*n* = 1152) from larger study named the Korean DEPression in ACS (K-DEPACS), 969 (84.1%) agreed to provide blood samples. There were no significant differences between those who agreed or not to provide blood samples with respect to any baseline characteristic (all *p*-values > 0.15). Of these 969 participants, depressive disorder (major or minor) at baseline was present in 378 (39.0%; major and minor depressive disorder as 177 (18.3%) and 201(20.7%), respectively). Demographic and clinical characteristics are compared by depressive disorder at baseline in Supplemental Table S1. Prevalent depressive disorder was significantly associated with female gender, lower educational level, living alone, rented housing, current unemployment, higher Hamilton Depression Rating Scale (HAMD) score, presence of hypertension and diabetes, and current smoking. Of the baseline participants, 255 participated in the Escitalopram for DEPression in ACS (EsDEPACS) trial (127 randomised to escitalopram and 128 to placebo), 49 (19%) of whom exited from the study after baseline, so the remaining 206 (104 on escitalopram and 102 on placebo) formed the sample for the 24 week treatment response analysis. Of all 969 participants at baseline, 711 (73.4%) were successfully followed. The 258 lost to follow-up had significantly older age and higher Killip class (*p*-values < 0.05) than those followed. At one year after ACS, incident depressive disorder was defined in 53 (12%) of the 426 participants without baseline depressive disorder; and persistent depressive disorder in 130 (46%) of the 285 participants with baseline depressive disorder.

**Table 1:** Plasma homocysteine concentrations and methylenetetrahydrofolate reductase (MTHFR) genotype by depressive disorder status at 2 weeks and at 1 year after acute coronary syndrome (ACS)

	Prevalent depressive disorder at 2 weeks after ACS			Incident depressive disorder at 1 year after ACS			Persistent depressive disorder at 1 year after ACS		
	Absent (*N*= 591)	Present (*N*= 378)	*p*-value	Absent (*N*= 373)	Present (*N*= 53)	*p*-value	Absent (*N*= 155)	Present (*N*= 130)	*p*-value
Homocysteine, mean (SD) μmol/l	10.2 (3.7)	12.0 (5.1)	<0.001	10.0 (3.7)	10.7 (4.3)	0.269	11.7 (4.8)	12.6 (5.9)	0.157
MTHFR genotype, N(%)									
C/C	214 (36.2)	128 (33.9)	0.749	136 (36.5)	18 (34.0)	0.740	51 (32.9)	41 (31.5)	0.882
C/T	277 (46.9)	185 (48.9)		177 (47.5)	28 (52.8)		73 (47.1)	65 (50.0)	
T/T	100 (16.9)	65 (17.2)		60 (16.1)	7 (13.2)		31 (20.0)	24 (18.5)	

*p*-value using t-tests or χ tests.

### Homocysteine concentration and MTHFR polymorphism by depression and remission status

Plasma homocysteine concentrations and MTHTR genotype are compared by depressive disorder status in Table 1. Prevalent depressive disorder was significantly associated with higher homocysteine concentration, while incident and persistent depressive disorder were not. In the 24 week drug trial, the overall remission rate was significantly higher in the escitalopram group (51.9%) compared to the placebo group (34.3%) (χ^2^ = 6.507, *p*-value = 0.011), as previously reported; however, homocysteine concentrations did not significantly differ by remission status in the total group or in the escitalopram or placebo group (1^st^ row of Supplemental Table S2). For MTHTR genotype, no deviation from the Hardy-Weinberg equilibrium was observed (*p*-values = 0.673), and no significant associations were found for MTHTR genotype with any depressive disorder or remission outcome (all *p*-values > 0.7). Logistic regression analysis for the associations of homocysteine concentrations and MTHFR genotype with depression and remission status after adjustment for covariates showed similar results (Table 2 and Supplemental Table S3).

**Table 2:** Multivariate analyses examining the interactive effects of homocysteine concentration and methylenetetrahydorfolate reductase (MTHFR) genotype on depression status

	Homocysteine concentration		MTHFR genotype		Homocysteine concentration X MTHFR genotype	
	Wald	OR (95% CI)	Wald	OR (95% CI)	Wald	OR (95% CI)
Prevalent depressive disorder^a^	30.63	1.11 (1.07-1.15)***	0.14	0.96 (0.79-1.18)	1.19	1.03 (0.98-1.08)
Incident depressive disorder^a^	0.51	1.03 (0.95-1.11)	0.05	0.95 (0.62-1.47)	3.65	1.10 (1.00-1.20)*
Persistent depressive disorder	1.13	1.04 (0.99-1.09)	0.17	0.93 (0.65-1.32)	3.90	1.12 (1.02-1.23)*

a adjusted for gender, education, living alone, housing, current employment, hypertension, diabetes, and current smoking

b adjujsted for the same model as in prevalent depressive disorder plus treatment status (escitalopram, placebo, and medical treatment only)

* *p*-value < 0.05

** *p*-value < 0.01

*** *p*-value < 0.001

### Interactive effects of homocysteine concentration and MTHFR genotype on depressive status

**Figure 1:**
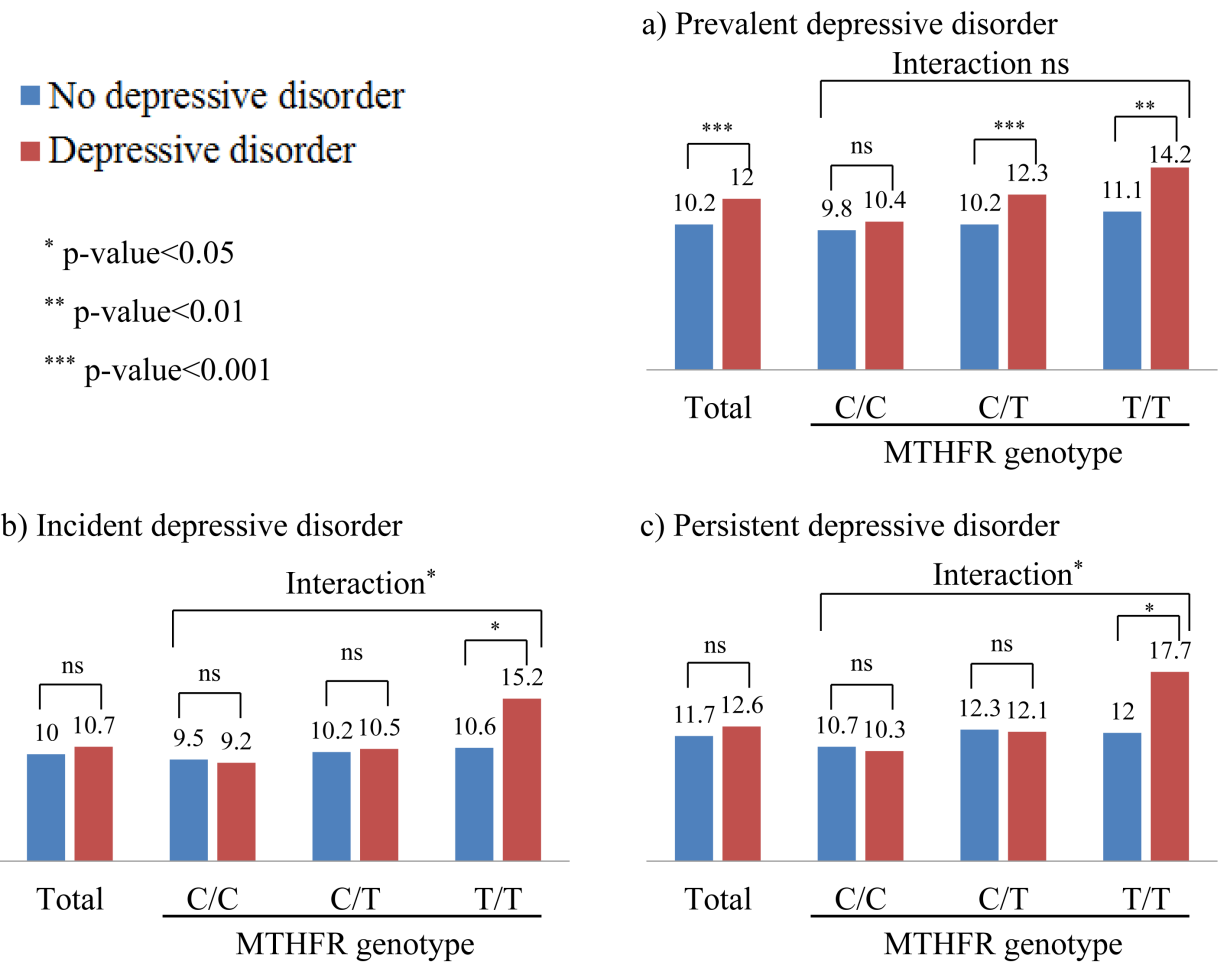
Plasma homocysteine levels by depressive disorder status and methylenetetrahydrofolate reductase (MTHFR) genotype Numeric data are plasma homocysteine level (μmol/l). *P*-values between depressive disorder status were drawn by logistic regression tests after adjustment for gender, education, living alone, housing, current employment, hypertension, diabetes, current smoking (prevalent and incident depressive disorder), plus treatment status (persistent depressive disorder). Interaction terms between homocysteine level and MTHFR genotype on depressive status were drawn in the same adjusted model.

Homocysteine levels were significantly associated with MTHFR genotype with mean (SD) levels 10.0 (3.5), 11.1 (4.1), and 12.3 (6.0) μmol/l for the C/C, C/T T/T genotypes respectively (F = 16.8, *p*-value < 0.001). Homocysteine concentrations are compared by depressive disorder status in each genotype in Figure 1. Higher homocysteine concentrations were significantly associated with prevalent depressive disorder in the presence of C/T and T/T genotypes, and with incident and persistent depressive disorder only in the presence of T/T genotype. Homocysteine concentrations are compared by remission status in each genotype in the 2^nd^ ∼ 4^th^ rows of Supplemental Table S2, but no significant associations were found. Interaction terms between genotype and raised homocysteine in logistic regression models after adjustment for covariates are summarized in 5^th^ ∼ 6^th^ columns of Table 2. Significant interactions were found for incident and persistent depressive disorder, but not for prevalent depressive disorder at baseline or for any marker of remission status.

### Associations with clinical hyperhomocysteinemia

**Figure 2:**
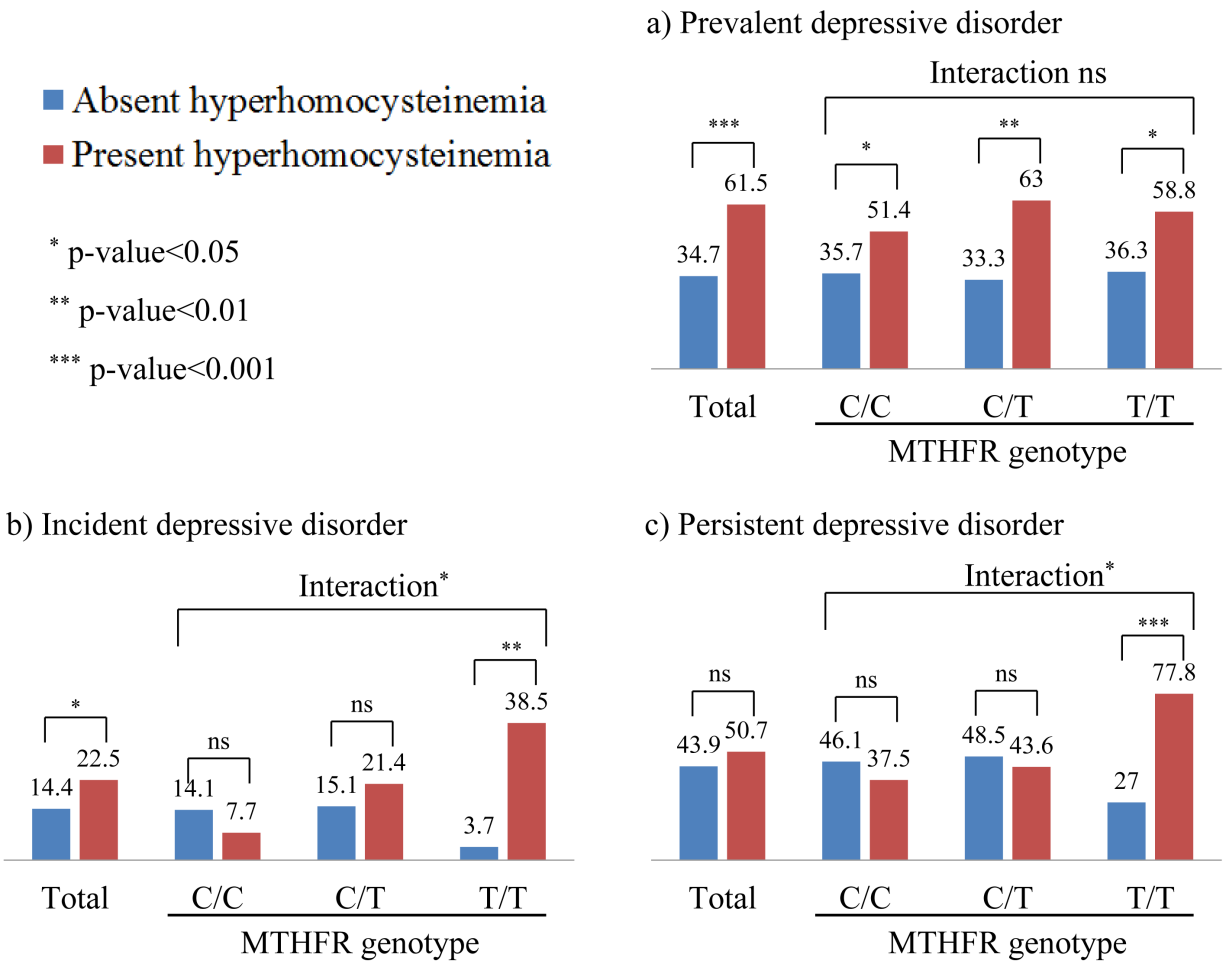
Depressive status by hyperhomocysteinemia and methylenetetrahydrofolate reductase (MTHFR) genotype Numeric data are percentages. *P*-values between absent and present hyperhomocysteinemia were drawn by logistic regression tests after adjustment for gender, education, living alone, housing, current employment, hypertension, diabetes, current smoking (prevalent and incident depressive disorder), plus treatment status (persistent depressive disorder). Interaction terms between hyperhomocysteinemia and MTHFR genotype on depressive status were drawn in the same adjusted model.

The prevalence of hyperhomocysteinemia at baseline was 16.1% (156 of 969 participants), and associations with depressive status are displayed in Figure 2. Overall, the findings were similar to those of continuously distributed homocysteine concentrations, but the strength of the associations was stronger in this categorical approach. In the total sample, hyperhomocysteinemia was significantly associated with prevalent (sensitivity 64%, specificity 63%) and incident depressive disorder, but not with persistent depressive disorder. When stratified by MTHFR genotype, hyperhomocysteinemia was significantly associated with prevalent depressive disorder in the presence of C/T and T/T genotypes, and with incident and persistent depressive disorder in the presence of T/T genotype (positive predictive value and negative predictive values for incident depressive disorder was 39 and 96%, respectively; and for persistent depressive disorder was 78% and 73%, respectively). Significant interactive effects of hyperhomocysteinemia and genotype were found for incident (*p*-value = 0.030) and persistent depressive disorder (*p*-value = 0.027), but not for prevalent depressive disorder (*p*-value = 0.243) with the same logistic regression model after adjustment. There were no significant individual or interactive effects of hyperhomocysteinemia and genotype on remission status in the 24 week trial (data not shown).

### Sensitivity analyses

Analyses were repeated after excluding participants reporting a history of previous depression (*N* = 14), and no substantial differences were found in any models (data not shown).

## DISCUSSION

Principal findings were independent associations between higher plasma homocysteine levels and depressive disorder after ACS, and differences in the pattern of this association by MTHFR genotype and time elapsed after ACS. Specifically, the cross-sectional association was significant regardless of MTHFR genotype shortly after ACS, but prospective associations were only significant in the presence of T allele for incidence and persistence of depressive disorder one year later. MTHFR C677T polymorphism was not itself associated with depressive disorder after ACS, and neither homocysteine levels nor MTHFR genotype were associated with 24 week antidepressant treatment responses in the nested trial of escitalopram.

Higher homocysteine levels and clinical hyperhomocysteinemia (above 15.0μmol/l) were significantly associated with prevalent depressive disorder shortly after ACS. Our findings are comparable to previous cross-sectional reports from unselected depressive patients [7, 17]. In addition, these results were irrespective of MTHFR genotype, and were not changed after excluding participants with a history of previous depression. Plausible explanations for this association were as follows. Homocysteine was found to be associated with dopamine metabolisim, serotonin deficiencies and oxidative stress which could be basis for depression occurrence [18, 19]. ACS related hyperhomocysteinemia [4] may predispose ACS patients to biologically vulnerable state for depressive disorders via theses various mechanisms and future research on the exact mechanisms is needed.

A primary role of the MTHFR genotype in the pathogenesis of depression related to ACS was not supported in the present study. However, it is noteworthy that MTHFR genotype significantly modified the associations of higher homocysteine levels with incident and persistent depressive disorder occurring in the chronic phase of ACS, although did not modify that with baseline depressive disorder. Plasma homocysteine levels were significantly higher in participants with higher numbers of T alleles, consistent with previous findings [6, 11, 12, 14]. T allele may confer a propensity to hyperhomocysteinemia which is sustained after the acute phase and acts on risk of depressive disorder over a longer period into the chronic phase of ACS, where general health and functional status become stronger determinants of mood state.

Our data on the diagnostic value of clinical hyperhomocysteinemia with MTHFR genotyping at baseline suggested meaningful clinical utility for depressive disorders. Negative predictive value of clinical hyperhomocysteinemia with T/T allele for incident depressive disorder at 1 year after ACS was excellent (96.3%) and positive and negative predictive value of those for persistent depressive disorder was modest (77.8 and 73.0%). Our results suggested that ACS patients with no hyperhomocysteinemia and C/C allele at acute phase were less likely to have depressive disorder newly at 1 year after ACS and depressive ACS patients with hyperhomocysteinemia and T/T allele at acute phase were more likely to have their depressive disorder at 1 year after ACS. Considering high prevalence of depressive disorder in ACS and usual measurement for homocysteine in cardiologic unit in practice due to importance of hyperhomocysteinemia as independent risk of ACS [20], plasma homocysteine assays with MTHFR genotyping could be utilized in the acute phase of ACS as screening to identify at-risk groups for depressive disorder at 1 year.

Notably, there were no significant associations of homocysteine levels or MTHFR genotype with 24-week escitalopram treatment responses evaluated in this study. Despite the interest in homocysteine and depression, modification of treatment response has received surprisingly little investigation. However, no association was found between homocysteine levels and time to clinical improvement with fluoxetine [15], or between C677T polymorphism and fluoxetine treatment response [16].

Our study has several strengths as the first evaluation of these questions. We were able to carry out a comprehensive evaluation of homocysteine and MTHFR genotype on the occurrence of depressive disorder in a large number of ACS patients with a prospective design. Additionally, a nested randomized placebo-controlled trial of the prospective study allowed investigation of influences on treatment response. Participants were recruited at baseline consecutively from all eligible patients with a recent ACS and were followed at 1 year thereafter, which reduced the risk of error arising from heterogeneous examination times and therefore increased the sample homogeneity. Depressive disorder was ascertained using a structured diagnostic interview, and all measurement methods for psychiatric and cardiovascular characteristics were well validated. Furthermore, a range of covariates were considered in the analyses. An important limitation is that homocysteine levels were only assayed at baseline and not at follow-up, since depression has been associated more strongly with changes in homocysteine levels over time [8]. Additionally, other psychiatric disorders such as post traumatic disorder was not investigated, which made it inconclusive whether homocysteine was a general marker of psychiatric risk given or whether homocysteine was specific to depression. There are also potential limitations arising from attrition in the recruitment process, and blood analysis was only possible in 84% of the baseline sample, although no differences were found in baseline demographic and clinical characteristics between those with and without this information. The 1-year follow-up was completed in 73% of whole baseline participants. The attrition was only associated with older age and poorer cardiac status, and was not associated with depressive status. It therefore appears that ACS patients with more severe baseline pathology were more likely to be lost to follow-up, which might obscure the associations of interest.

Our results support a role of homocysteine in the pathogenesis of depressive disorder comorbid with ACS independently at acute phase and interactively with MTHFR genotype at chronic phase, and have several potential implications. Plasma homocysteine assays, particularly for clinical hyperhomocysteinemia, might thus have modest clinical utility of screening to identify at-risk groups for depressive disorder in the acute phase of ACS. Our findings may also aid to identify suitable therapeutic interventions. That is, depression comorbid with ACS also leads to very high disease burden, and is difficult to treat [21, 22], and assaying homocysteine and MTHFR genotype might allow more focused interventions for the prevention or management of depressive disorder in the late phase of ACS in more susceptible sub-groups. Trials of long term vitamin supplementation on depressive disorder have been controversial to date [23-25], but based on our findings, supplement effects might be more prominent in those with MTHFR TT genotype, at least in ACS patients. Relationships with the dose, duration and, particularly, constituents of vitamin supplements should therefore be investigated.

## MATERIALS AND METHODS

### Study outline and participants

Data were drawn for analysis from larger study named the Korean DEPression in ACS (K-DEPACS) study, which also contained a nested randomized controlled trial, the Escitalopram for DEPression in ACS (EsDEPACS) study. The overall design has been described in detail [26], and the recruitment process is summarized in Supplemental Figure S1. The K-DEPACS study was carried out from 2006 to investigate the epidemiology of depression in ACS using a observational prospective design. Participants were consecutively recruited from patients recently hospitalized with ACS (*N* = 4809) at the Department of Cardiology of Chonnam National University Hospital, Gwangju, South Korea. Patients were treated based on international guidelines for the management of ACS [27] by the study cardiologists. Those who met eligibility criteria and agreed to participate (*N* = 1152) were assessed for a depressive disorder diagnosis by the study psychiatrists using the Mini-International Neuropsychiatric Interview (MINI) [28] as inpatients within 2 weeks post-ACS, and thereafter as outpatients every 4 weeks up to 12 weeks. Of these, 969 agreed to blood assays and comprised the baseline sample. Of 378 patients with depressive disorder in this sample, 255 also agreed to participate in a 24-week, double-blind, nested randomized placebo-controlled trial of escitalopram efficacy and safety: the EsDEPACS study (ClinicalTrial.gov registry number: NCT00419471). The first patient was enrolled in May 2007 and the last patient completed the follow-up evaluation in March 2013. Examinations were scheduled at baseline, and weeks 4, 8, 12, 16, 20, and 24 thereafter. The details and main results of this trial have been published, in which escitalopram was found to be superior to placebo in reducing depressive symptoms [29]. The remaining 123 patients who declined participation in the trial received conventional medical treatment for ACS only. Detailed eligibility of the K-DEPACS and EsDEPACS studies were described in Supplementary Materials. All participants in the K-DEPACS and EsDEPACS studies were approached for a re-examination of depressive status at 1 year after the baseline evaluation. Written informed consent was collected for the K-DEPACS and EsDEPACS studies, both of which were approved by the Chonnam National University Hospital Institutional Review Board.

### Evaluations for depressive status

Diagnoses of depressive disorder were determined using the MINI, a structured diagnostic psychiatric interview for DSM-IV defining major or minor depressive disorder categories as outputs [27]. Due to the distinctive study design at baseline, symptom- duration criteria was within rather than at least 2 weeks. Depressive disorder was defined as the combined category, since there were insufficient numbers with major depressive disorder to analyze separately. Based on the evaluation at two phases after ACS, depressive disorder was ascertained as baseline prevalent, and follow-up incident (cases at follow-up within the sample who did not have depressive disorder at baseline) or persistent (cases at follow-up within the sample who did have depressive disorder at baseline). In the 24-week EsDEPACS trial, remission status was evaluated at each follow-up point, and defined on the basis of a HAMD score ≤7.

### Plasma homocysteine and MTHFR C677T polymorphism

Blood samples were collected in a fasting state and were carried out in the mornings where possible. They were drawn into EDTA tubes, and centrifuged, separated into plasma aliquots, and stored at −80°C within 2 hours of collection. Total plasma homocysteine concentration was measured using commercially available (AxSYM Homocysteine Reagent Pack, Abbott Laboratories, USA) high-performance liquid chromatography. MTHFR C677T genotype was determined by a polymerase chain reaction (PCR) and HinFI restriction enzyme digestion as described previously^8^ with miner modification. HinfI digestion (1.5U/25μL reaction mixture) was performed directly in the PCR tube at 37°C for 4 hours before analysis of restriction fragments by polyacrylamide gel electrophoresis.

### Demographic and clinical covariates

Characteristics potentially acting as confounding or mediating factors for associations between depressive disorder and ACS were evaluated at baseline. Data on age, gender, duration of education, living status (living alone or not), accommodation tenure (owned or rented), current occupation (employed or not), and previous and family histories of depression were obtained. The following cardiovascular risk factors were ascertained: previous and family histories of ACS, diagnosed hypertension and diabetes mellitus, hypercholesterolemia by fasting serum total cholesterol level (>200mg/dL), obesity by measured body mass index (BMI), and reported current smoking status. For current cardiac status, severity of ACS was estimated by the Killip classification [30], left ventricular ejection fraction (LVEF) was estimated using echocardiography, and serum cardiac biomarkers on troponin I and creatine kinase-MB (CK-MB) were measured. Other factors can affect the homocysteine concentrations were considered: serum creatinine level and vitamin supplementation.

### Statistical analyses

Demographic and clinical characteristics were compared between patients with and without prevalent depressive disorder at baseline using t-tests or χ^2^ tests, as appropriate. Characteristics significantly associated with depressive disorder (*P* < 0.05) were used as covariates in further regression models. Plasma homocysteine was first considered as a continuous value. Homocysteine concentration and MTHFR C677T polymorphism were compared between those with and without prevalent/incident/persistent depressive disorder using t-tests and χ^2^ tests, respectively. In the 24-week EsDEPACS trial, homocysteine concentration and MTHFR C677T polymorphism were compared between those who did or did not achieve remission using t-tests and χ^2^ tests, respectively. Odds ratios (ORs) for depression and remission status were estimated using logistic regression models, adjusting for relevant covariates. To investigate the potential interactive effects of homocysteine concentration and MTHFR C677T polymorphism on depressive status and remission status, following analyses were carried out: i) homocysteine concentration was compared between genotypes using ANOVA, ii) associations between homocysteine concentration and depressive status were evaluated in each genotype; iii) two way interactions between homocysteine concentration and genotype were tested using multivariate logistic regression models. Additional analyses were carried out to investigate associations with a clinically significant category of hyperhomocysteinemia defined as a plasma level above 15.0 μmol/l [31] with the same analytic methods above. Additionally, diagnostic statistics namely, sensitivity, specificity, positive predictive value and negative predictive values were calculated for depressive disorder status.

Finally, sensitivity analyses were carried out with the same analytic methods after excluding participants with a previous history of depression. Statistical analyses were carried out using SPSS 18.0 software.

## SUPPLEMENTARY MATERIALS METHODS, FIGURE AND TABLES


