# Supplementary material for: Predictive value of homocysteine for depression after acute coronary syndrome
Source: Oncotarget. 2016 Sep 10;7(42):69032–40. doi: 10.18632/oncotarget.11966 (PMC5356609; doi:10.18632/oncotarget.11966)
Supplement: Supplementary file 1 [file oncotarget-07-69032-s001.pdf]

Predictive value of homocysteine for depression after acute coronary syndrome

Supplemental Information

SUPPLEMENTAL METHODS……………………………………………………………..2

Eligibility……………………… ………………………………………………………..2

SUPPLEMENTAL FIGURE S1…..………….……………………………………………..4

SUPPLEMENTAL TABLE S1…..……………….………………………………………..5

SUPPLEMENTAL TABLE S2…..……………….………………………………………..7

SUPPLEMENTAL TABLE S3…..……………….………………………………………..8

SUPPLEMENTAL METHODS

Eligibility Criteria

Of all patients who visited the study site with angina symptoms resulting in hospitalization, inclusion criteria for K-DEPACS study entry were as follows: i) aged 18~85 years; ii) confirmed ACS by investigation (the presence of ST-segment elevation MI was determined by >30 min of continuous chest pain, a new ST-segment elevation ≥2 mm on at least two contiguous electrocardiographic leads, and creatine kinase-MB more than three times normal; the presence of non-ST-segment elevation MI was diagnosed by chest pain and a positive cardiac biochemical marker without new ST-segment elevation; and the presence of unstable angina was determined by chest pain within the preceding 72 h with or without ST-T wave changes or positive cardiac biochemical markers); iii) ability to complete study questionnaires; iv) ability to understand the study objectives and sign informed consent. Exclusion criteria were: i) occurrence of ACS while hospitalized for another reason; ii) ACS developing less than 3 months after a coronary artery bypass graft procedure; iii) uncontrolled hypertension (systolic blood pressure (BP) >180mmHg or diastolic BP >100mmHg); iv) resting heart rate <40/min; v) severe physical illnesses threatening life or interfering with the recovery from ACS; vi) persistent clinically significant laboratory abnormalities in complete blood cell counts, thyroid or renal function tests. For the EsDEPACS trial, additional inclusion criteria were as follows: i) Beck Depression Inventory (BDI)[1] >10; ii) major or minor depressive disorder according to DSM-IV criteria. Additional exclusion criteria were: i) concomitant use of class I antiarrhythmic medications, reserpine, guanethidine, clonidine, methyldopa, lithium, anticonvulsants, antipsychotics, or antidepressants; ii) history of neuropsychiatric illnesses such as dementia, Parkinson's disease,

brain tumor, psychosis, bipolar disorder, alcoholism, or other substance dependence; iii) pregnancy; iv) participating in other drug trials.

Reference

1. Beck AT, Ward CH, Mendelson M, Mock J, Erbaugh J. An inventory for measuring depression. Arch Gen Psychiatry 1961;4:561-571.

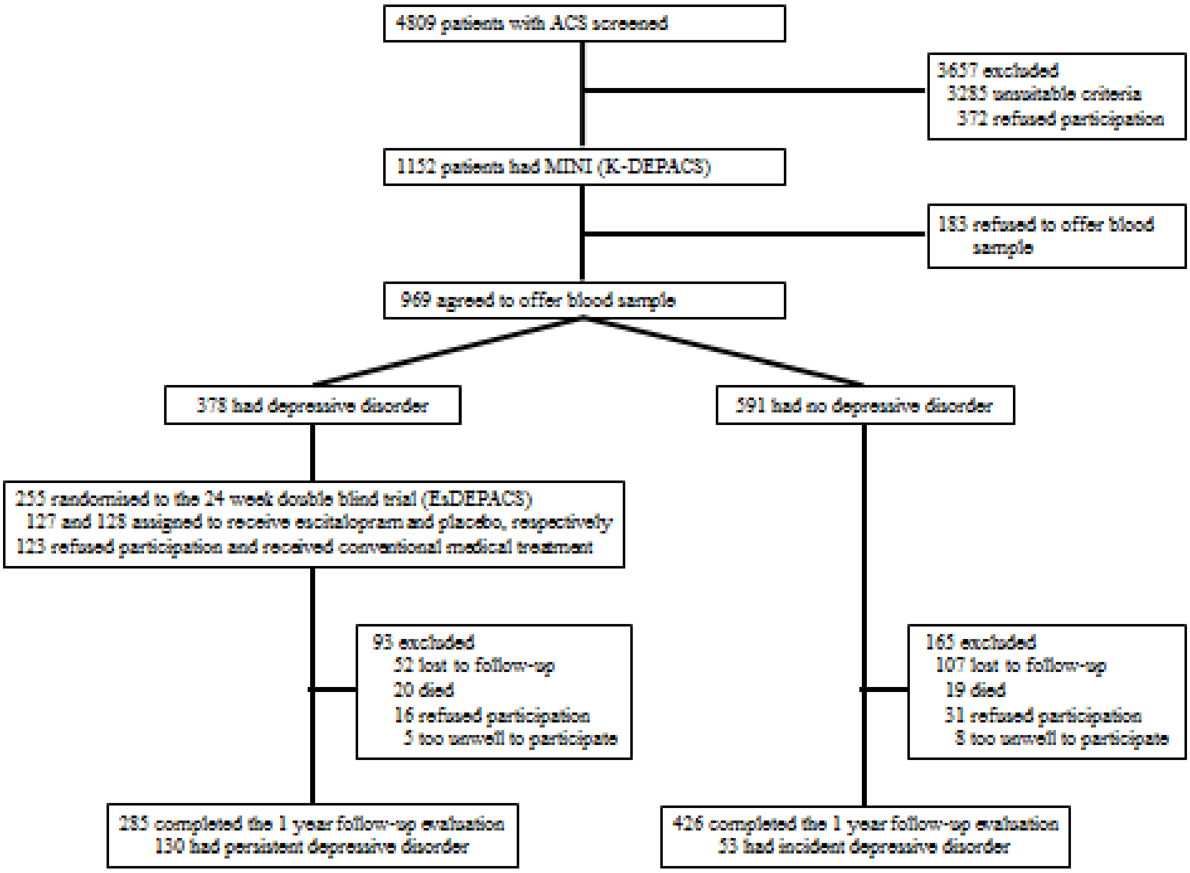

Supplemental Figure S1: Flow diagram for the recruitment process, ACS, acute coronary syndrome; MINI, Mini-International Neuropsychiatric Interview. K-DEPACS, Korean DEPression in Acute Coronary Syndrome study; EsDEPACS, Escitalopram for DEPression in Acute Coronary Syndrome study.

Supplemental Table S1: Sample characteristics by baseline depressive disorder status

	No depressive disorder (N=591)	Depressive disorder (N=378)	Statistical coefficient	P-value*
**Socio-demographic characteristics**				
Age, mean (SD) years	57.7 (11.3)	59.0 (10.8)	t=-1.860	0.063
Gender, N (%) female	118 (20.0)	151 (39.9)	χ^2=45.90	**<0.001**
Education, mean (SD) years	10.2 (4.8)	9.3 (4.4)	t=+3.013	**0.003**
Living alone, N (%)	47 (8.0)	45 (11.9)	χ^2=4.191	**0.041**
Housing, N (%) rented	73 (12.4)	77 (20.4)	χ^2=11.33	**0.001**
Currently unemployed, N (%)	192 (32.5)	176 (46.6)	χ^2=19.39	**<0.001**
**Depression characteristics**				
Previous depression, N (%)	17 (2.9)	17 (4.5)	χ^2=1.789	0.181
Family history of depression, N (%)	11 (1.9)	12 (3.2)	χ^2=1.716	0.190
HAMD, mean (SD) score	3.6 (2.7)	14.2 (5.0)	t=-38.09	**<0.001**
**Cardiac risk factors**, N (%)				
Previous ACS	20 (3.4)	19 (5.0)	χ^2=1.610	0.205
Family history of ACS	15 (2.5)	16 (4.2)	χ^2=2.138	0.144
Hypertension	252 (42.6)	206 (54.5)	χ^2=13.01	**<0.001**
Diabetes mellitus	93 (15.7)	98 (25.9)	χ^2=15.13	**<0.001**
Hypercholesterolemia	296 (50.1)	190 (50.3)	χ^2=0.003	0.956
Obesity	259 (43.8)	156 (41.3)	χ^2=0.614	0.433
Current smoker	249 (42.1)	117 (31.0)	χ^2=12.26	**<0.001**

**Current cardiac status**				
Killip class >1, N (%)	97 (16.4)	71 (18.8)	χ^2=0.904	0.342
LVEF, mean (SD) %	61.4 (11.2)	60.8 (11.4)	t=+0.772	0.440
Troponin I, mean (SD) mg/dL	9.9 (16.6)	9.9 (11.8)	t=+0.063	0.949
CK-MB, mean (SD) mg/dL	17.6 (41.1)	17.1 (30.4)	t=+0.178	0.858
**Other factors**				
Creatinine, mean (SD) mg/dl	0.9 (0.3)	0.9 (0.3)	t=+1.013	0.462
Vitamin supplement, N (%)	13 (2.2)	10 (2.6)	χ^2=0.198	0.657

*p-values using t-tests or χ^2 tests as appropriate.

HAMD, Hamilton Depression Rating Scale; ACS, acute coronary syndrome; LVEF, left ventricular ejection fraction; CK-MB, Creatine kinase-MB.

Supplemental Table 2 Plasma homocysteine mean (SD) µmol/l concentrations by remission, methylenetetrahydorfolate reductase (MTHFR) genotype, and treatment drug in the 24 week trial

	Total			Escitalopram			Placebo		
	No remission (N=117)	Remission (N=89)	p-value	No remission (N=50)	Remission (N=54)	p-value	No remission (N=67)	Remission (N=35)	p-value
Total group	12.3 (5.0)	11.4 (3.6)	0.192	12.2 (6.4)	11.5 (4.0)	0.493	12.3 (3.7)	11.3 (3.0)	0.190
MTHFR genotype									
C/C	10.7 (3.3)	10.6 (3.2)	0.908	9.6 (3.3)	10.4 (3.6)	0.694	11.3 (3.3)	11.0 (2.4)	0.752
C/T	12.3 (4.2)	12.1 (4.3)	0.798	12.6 (4.9)	12.6 (4.7)	0.994	12.1 (3.7)	11.3 (3.8)	0.502
T/T	14.6 (7.8)	11.6 (1.9)	0.141	15.2 (11.7)	11.4 (2.3)	0.350	14.2 (3.8)	11.9 (1.3)	0.145

p-value using t-tests.

Supplemental Table 3 Multivariate analyses examining the interactive effects of homocysteine concentration and methylenetetrahydorfolate reductase (MTHFR) genotype on remission status

	Homocysteine concentration		MTHFR genotype		Homocysteine concentration X MTHFR genotype	
	Wald	OR (95% CI)	Wald	OR (95% CI)	Wald	OR (95% CI)
**Remission status**						
Total group[a]	1.32	0.96 (0.89-1.03)	0.39	0.88 (0.58-1.33)	0.87	0.94 (0.83-1.07)
Escitalopram group[a]	0.81	0.96 (0.87-1.05)	0.45	0.81 (0.44-1.50)	0.26	0.96 (0.82-1.13)
Placebo group[a]	0.90	0.93 (0.81-1.08)	0.01	1.01 (0.53-1.92)	0.54	0.92 (0.74-1.15)

[a]adjusted for gender, education, living alone, housing, current employment, hypertension, diabetes, and current smoking
